# Increased risk of deep venous thrombosis in patients with poor ankle dorsiflexion after lower limb immobilization

**DOI:** 10.1097/OI9.0000000000000038

**Published:** 2019-05-17

**Authors:** Susanna Aufwerber, Praxitelis Praxitelous, Gunnar Edman, Karin Grävare Silbernagel, Paul W. Ackermann

**Affiliations:** a Department of Molecular Medicine and Surgery, Karolinska Institutet; b Functional Area Occupational therapy and Physiotherapy, Allied Health Professionals Function; c Department of Orthopedic Surgery, Karolinska University Hospital, Stockholm; d Department of Psychiatry, Tiohundra AB, Norrtälje, Sweden; e Department of Physical Therapy, University of Delaware, Newark, DE.

**Keywords:** achilles tendon, deep venous thrombosis, immobilization, patient-reported outcome measures, plaster cast, rupture

## Abstract

**Objective:**

Many trauma patients are at risk of pulmonary embolism due to unrecognized deep vein thrombosis (DVT). Restricted ankle dorsiflexion (AD) range of motion during leg immobilization is known to cause reduced venous blood flow. The aim of the present study was to assess whether AD at plaster cast removal is related to the incidence of DVT and to patient outcome.

**Design:**

Prospective observational cohort study.

**Setting:**

Level 1 Trauma Center.

**Patients:**

A total of 124 patients (97 men, 27 women; mean age 40.3 years) with plaster cast leg immobilization after surgical repair of Achilles tendon rupture were assessed.

**Main outcome measures:**

At 2 weeks postoperatively, assessments of AD and the incidence of DVT using compression duplex ultrasound were performed with observers blinded to patient grouping. Patients were dichotomized into 2 groups; poor or good AD, according to the mean AD, −7°. At 3- and 12 months patient-reported outcome was examined using validated questionnaires (ATRS and FAOS), and functional outcome using the heel-rise test.

**Results:**

Patients with poor AD sustained 42% DVTs, while patients with good AD exhibited a DVT-rate of 23% (*P* = .036). Logistic regression analysis corroborated this finding (OR = 2.62, *P* = .036; 95% CI = 1.06–6.44). AD was not linked to any long-term functional or patient-reported outcome.

**Conclusions:**

Reduced AD after plaster cast removal is associated with a higher risk of DVT. The results of this observational study warrant further prospective studies to confirm the effects of ankle dorsiflexion on the risk of developing venous thromboses.

**Level of evidence:** II

## 1 Introduction

Lower limb immobilization, such as after trauma or surgery, is associated with a high risk (up to 40%) of DVT, according to a meta-analysis.^[1]^ The rate of DVT in patients with leg immobilization after Achilles tendon rupture (ATR) has been reported as high as 50%.^[2–5]^ Most of these DVTs are, however, asymptomatic and not diagnosed at plaster cast removal. Thus, at present, predictive factors and markers relating to the risk of DVT during limb immobilization are lacking.^[6]^

To evaluate the probability of DVT the Wells score is used, where a score of ≥2 indicates that DVT is likely and that the patient should undergo a diagnostic ultrasound scan.^[7]^ Asymptomatic patients, with lower limb immobilization, generally only receive a score of 1 if there is no calf swelling. Recently, however, asymptomatic DVTs were demonstrated to exhibit an up to 72% risk of pulmonary embolism.^[8]^ Therefore, investigating other signs of DVT during leg immobilization is important for prevention as well as risk assessment.

A recent study demonstrated that ankle dorsiflexion exercises during limb immobilization induced 2- to 6-fold increases in blood velocity in the popliteal vein.^[9]^ Moreover, previous studies on leg immobilization have indicated that fixation in plantar flexion was associated with a significant reduction in venous blood flow as compared with the ankle in a neutral position.^[10]^ It is therefore plausible that plaster casts fixed in more plantar flexion, i.e., less AD results in more venous stasis and thereby increased risk of blood clot formation.

DVT during leg immobilization was recently shown to be associated with poor patient outcome 1 year after acute ATR.^[11]^ A recent meta-analysis comparing early postoperative ankle motion and weightbearing, with traditional ankle immobilization with a non-weightbearing rigid cast, demonstrated superior patient-reported outcomes and faster return to prior sporting activities in the weightbearing group.^[12]^ However, immobilization in a non-weightbearing plaster cast is still widely used postoperatively. Thus, whether decreased AD, per se, after immobilization is related to impaired patient-reported outcome after ATR has been unknown.

In the present study, it was therefore hypothesized that AD after the removal of a non-weightbearing plaster cast would be associated with the risk of DVT as well as patient outcome after surgical repair of ATR. The primary aim of the study was to assess AD in relation to DVT. The secondary aim was to assess if AD was associated with patient-reported outcome at 3 and 12 months postoperatively as well as functional outcome at 1 year.

## 2 Methods

Ethical approval was obtained from the Regional Ethical Review Committee in (Stockholm, Sweden), (Dnr: 2009/2079-31/2 and Dnr: 2013/1791-31/3).

### 2.1 Patients

Between March 2011 and February 2018, a total of 124 plaster-casted control-patients, without any intervention, from 2 different randomized controlled trials were eligible for analysis in the study (Table 1). All participants received oral and written information about the study procedure and provided written informed consent prior to surgery. Patients between 18 and 75 years with an acute unilateral Achilles tendon rupture, who had surgery within a week from injury, were eligible for inclusion in the randomized clinical trial and exclusion criteria were as earlier published.^[3]^ Preoperatively all patients received crutches and were advised to be non-weightbearing, and were immobilized either in an elastic wrap or in a plaster back slab.

**Table 1:**
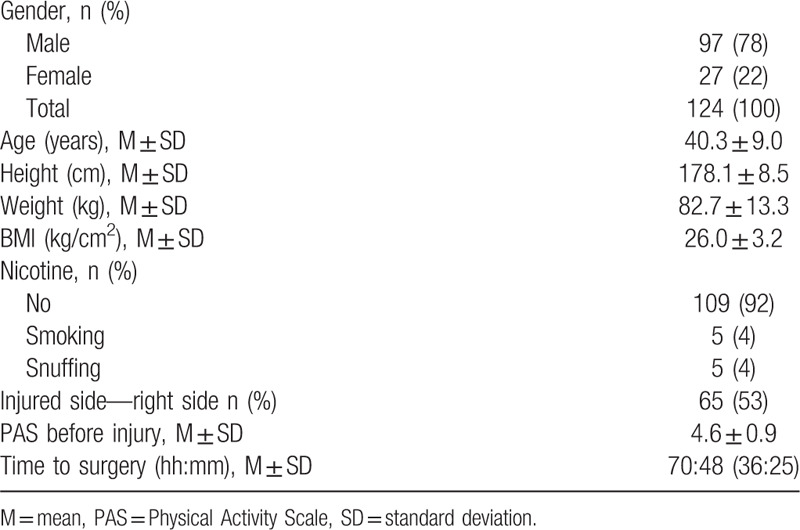
Baseline demographic characteristics

### 2.2 Surgical procedure

A standardized surgical procedure was performed, using local anesthesia and the modified Kessler suture technique, on an outpatient basis as described earlier.^[3]^

### 2.3 Postoperative regime

The patients received a standard below-knee plaster cast with the ankle in approximately 30 degrees equinus position shortly after the completion of surgery. The plantar flexion in the plaster cast was, however, not measured so that there may be an actual small variation in plantar flexion, which constitutes the basis for this study. Patients were non-weightbearing during the first 2 postoperative weeks. Patients were recommended to perform active toe movements and thigh exercises according to a rehab protocol during the first 2 weeks. No antithrombotic prophylactic drugs were given to the patients postoperatively. At 2 weeks postoperatively, the plaster cast was removed, and the patient was sent for the assessment of ankle dorsiflexion, calf circumference, and DVT screening.

### 2.4 Evaluations at the 2-week plaster cast removal

#### 2.4.1 DVT screening

All patients were screened for DVT in the injured leg by unilateral compression duplex ultrasound (CDU). A trained nurse and 2 experienced ultrasonographers, blinded to the outcome of the other tests, performed all the CDU scans using a Philips CX 50 ultrasound machine (Philips Medical Systems, Andover, Massachusetts). The standard procedure included evaluation of all deep proximal and distal veins as previously described.^[3]^

Most DVTs found were asymptomatic. The exact number of asymptomatic/symptomatic DVTs was not further assessed since earlier studies have demonstrated difficulties in differentiating symptoms of DVT from normal postoperative findings.^[3,4]^ Patients diagnosed with a DVT received anticoagulation treatment according to the protocol at the hospital. One patient exhibited a proximal DVT and 1 patient developed a symptomatic pulmonary embolism diagnosed with computer tomography. In this study, we choose not to perform screening of the contralateral nonimmobilized limb with ultrasound Doppler, since this was not within the scope of this article.^[13]^

#### 2.4.2 Ankle dorsiflexion range of motion assessment

AD was in this study defined as the maximum ankle dorsiflexion range of motion the patient could perform and was assessed at 2 weeks postoperatively. An experienced physiotherapist, blinded to the DVT examination, performed all AD assessments. After removal of the plaster cast, the patient was first asked to perform 10 repetitions of active plantar-/dorsiflexion movements with both feet. For testing, the patient was sitting with his/her feet in full contact on the ground and was asked to slide the foot backward as far as tolerated without lifting the heel off the floor. The patient was instructed to slide backward with the injured leg as much as possible but without inducing pain or discomfort. A goniometer parallel to the floor and the fibula was used to record the amount of maximal ankle dorsiflexion range of motion. Each leg was measured 3 times and an average degree of dorsiflexion was calculated. Both ankles were examined separately, with the healthy side tested first. For analysis, AD was dichotomized according to the mean value (M = −7; “Poor” = ≤ −7 vs “Good” = > −7, to provide 2 groups. An AD of −7 equals 7 degrees short of neutral, that is, in plantar flexion.

#### 2.4.3 Calf circumference measurement

Calf circumference was measured at the thickest part of the lower leg with a tape measure with the patient sitting with thighs supported and lower legs hanging free.

### 2.5 Three and 12-months follow-up evaluations

#### 2.5.1 Patient-reported outcome measure (PROM)

Patient-reported outcome was assessed with self-administered questionnaires specific for symptoms from the foot and ankle. The Achilles tendon Total Rupture Score (ATRS) consists of 10 items and responses ranging from 0 (“Worst”) to 10 (“Best”).^[14]^ A total score of 100 is presented. A lower score indicates aggravated symptoms and greater limitation on physical activity. The Foot and Ankle Outcome Score (FAOS) consists of 41 items, evaluating patient symptoms and pain, foot- and ankle function and quality of life.^[15]^ The questionnaire is divided into 5 subscales, and the score ranges from 0 (“Worst”) to 100 (“Best”).

The physical activity score evaluates and compares the patient physical activity before and after the injury. It is scored from 1 (no physical activity) to 6 (heavy physical exercise several times/week).^[16]^

The ATRS was completed at 3 months (n = 62) and 12 months (n = 95) and the FAOS at 12 months (n = 94). For the analysis of the ATRS at 3 months, only the fourth item (pain) was used. Physical activity score was assessed both pre- (n = 103) and postinjury (n = 90).

#### 2.5.2 Heel-rise test

Muscular endurance testing was conducted at 12 months postoperatively on both limbs. The MuscleLab (Ergotest Technology, Oslo, Norway) measurement system was used for the evaluations and was performed as previously described in the literature.^[11,17]^ The Limb Symmetry Index (LSI = (injured/uninjured) × 100) was calculated for heel-rise height, repetitions, and total work.

### 2.6 Statistical analysis

Recent studies have demonstrated the rate of compression Doppler ultrasound verified DVTs after ATR surgery to be 40%. Based on earlier studies of ankle dorsiflexion we estimated a 60% risk reduction of the incidence of DVT between patients with poor as compared with good ankle dorsiflexion. Thus, 54 patients per group would be required to detect a difference in DVT with 80% power when alpha was set equal to 5%.

Descriptive data were reported as mean, standard deviation, and frequency. All variables were checked for severe skewness. Pearson correlations coefficient was used for correlations between variables and outcome for normally distributed data. The correlations were verified with multiple linear regressions analyses (stepwise forward method, with an inclusion level of 0.05). This was done to investigate the unique relationships between the independent variables [gender, age, nicotine, body height, weight and body mass index (BMI), DVT] and the dependent variable. Logistic regression analysis was performed to investigate the relationship between DVT and AD. All data were analyzed in SPSS version 22 (IBM SPSS, Version 22.0. Armonk, New York). The level of significance was ≤5% for all analyses.

## 3 Results

### 3.1 Ankle dorsiflexion range of motion (AD)

The ankle dorsiflexion range of motion (AD, degrees of dorsiflexion) at 2 weeks’ post leg immobilization was significantly lower (*P* < .001) in the immobilized, injured- [M ± SD = −6.9 ± 6.4; range: −21 to 13 degrees] compared with the contralateral noninjured limb [M ± SD = 29.7 ± 5.4; range: 9–40] (Table 2). The patients were dichotomized into 2 groups (poor and good AD) according to the mean AD, −7 degrees. The 2 groups were studied in relation to age, gender and BMI (below or above 26). Male gender was found significantly associated with poorer AD (age: χ^2^ = 0.047, *P* = .828; gender: χ^2^ = 4.15, *P* = .042; and BMI: χ^2^ = 0.51, *P* = .477).

**Table 2:**
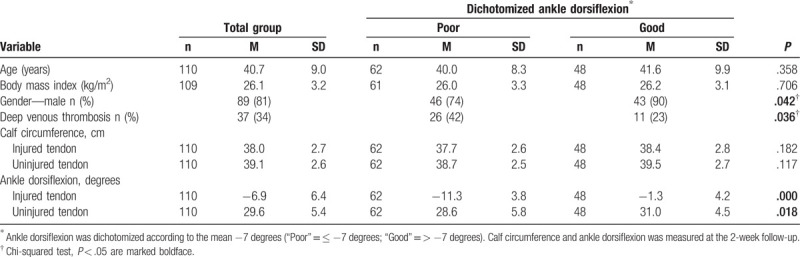
Baseline demographics and clinical measures for patients with ankle dorsiflexion data (n = 110)

### 3.2 Deep venous thrombosis (DVT)

After 2 weeks of lower-limb immobilization, the overall incidence of DVT was 33% (40/121). 42% (26/62) of the patients with poor AD sustained a DVT, while only 23% (11/48) of the patients with good AD suffered a DVT (*P* = .036) (Table 2).

The relationship between dichotomized AD and DVT was further controlled in a logistic regression analysis also including gender and dichotomized age. The DVT rate was significantly associated with AD and increased age, independently of each other. The odds ratio of sustaining a DVT at 2 weeks of immobilization was 2.62 in patients with poor AD and 3.98 in patients with age above 40 (Table 3).

**Table 3:**
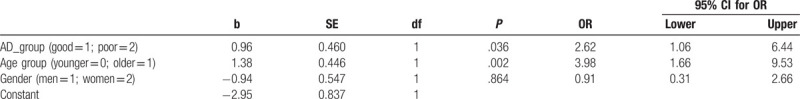
Relationships between ankle dorsiflexion, gender and age, and incidence of DVT at 2 weeks

### 3.3 Calf circumference

The calf circumference of the patients at 2 weeks of immobilization was neither significantly associated with AD (*P* = .952) nor with the risk of exhibiting a DVT (*P* = .317).

### 3.4 Patient-reported outcome at 3 and 12 months

Patients with poor AD exhibited at 3 months significantly lower scores of the ATRS subscale “Pain in the calf/tendon/foot” (M = 7.9 vs 6.6; *P* = .045) (Table 4). The occurrence of a DVT, however, was not correlated with “Pain in the calf/tendon/foot” (r = −0.195 *P* = .11). Multiple linear regression corroborated that decreased AD was significantly associated with more pain in the limb when taking DVT, age, gender, and BMI into consideration [F(1, 63) = 4.169, *P* = .045, R^2^ = 0.062].

**Table 4:**
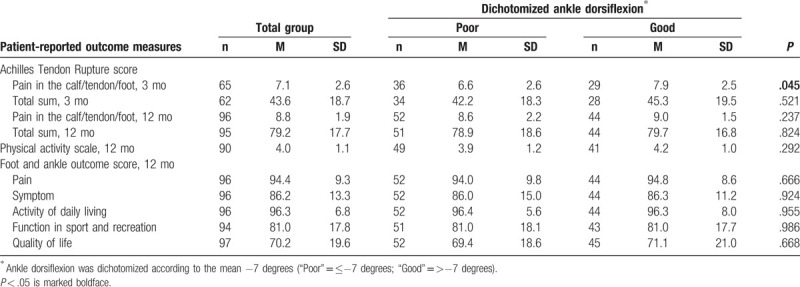
Patient-reported outcome measures

Self-reported outcome at 12 months was studied with 2 inventories—ATRS and FAOS. There were at 1 year no significant differences in any of the subscales or the total scales of ATRS and FAOS between patients with poor and good AD (Table 4).

### 3.5 Physical activity level at 12 months

There was no significant difference between the patients with regards to AD in self-reported physical activity before injury (*P* = .226) or at 12 months’ follow-up (*P* = .292) (Table 4).

### 3.6 Functional outcome at 12 months

Functional outcome was measured at 12 months with the heel-rise test—the limb symmetry index (LSI) of the number of repetitions, LSI of the heel-rise height, and total work. There were no significant differences between the patients with poor or good AD in any of the functional tests (Table 5).

**Table 5:**
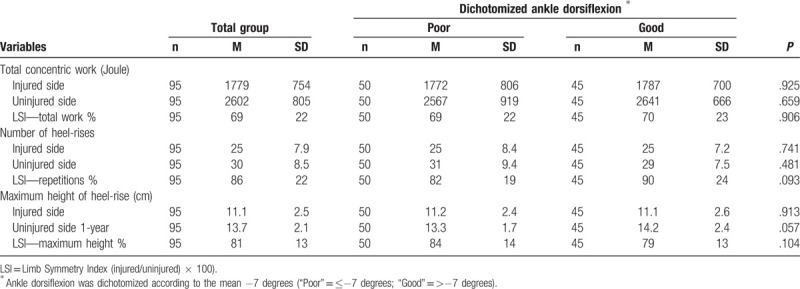
Heel-rise test at 1 year postoperatively

## 4 Discussion

The present study established that patients with Achilles tendon rupture surgery who exhibited reduced ankle dorsiflexion during plaster cast removal after leg immobilization were associated with an increased risk of suffering a DVT. The calf circumference, however, was not related to the incidence of DVT. Moreover, decreased ankle dorsiflexion was related to more pain in the limb at 3 months postoperatively, but without any signs of long-term adverse events in terms of functional and patient-reported outcome.

When exploring a potential causal link between ankle dorsiflexion and the risk of DVT it may be useful to review the risk factors responsible for the development of a thrombosis, which according to Virchow's triad are hypercoagulability, endothelial damage, and venous stasis.^[18]^ While hypercoagulability and endothelial damage are presumably not related to ankle dorsiflexion during immobilization, it is more likely that ankle positioning and mobility during plaster casting may affect venous stasis. The observed decreased ankle dorsiflexion among half of the limb immobilized patients, which were more prone to develop DVT, may suggest that their plaster casts were fixed in more plantar flexion due to, for example, that the Achilles tendon was surgically fixed in more plantar flexion or genetic differences, allowing less ankle mobility and less venous return. Increased plantar flexion (equinus) and more rigidity of the lower limb during immobilization may be 2 factors, which according to recent studies could increase both venous stasis and the risk of DVT.^[9,10]^

The most important finding in this study thus pertained to establishing that patients with greater ankle dorsiflexion during limb immobilization exhibited half the risk of sustaining a DVT as compared with patients with lesser ankle dorsiflexion. Such a difference in the risk of sustaining a DVT suggests that it may be crucial to find other interventions to increase circulation during leg immobilization to reduce the risk of venous thromboembolism.^[19]^

An earlier study suggested that leg immobilization in a walking boot with the ankle in neutral compared to equinus position significantly increased venous return.^[10]^ Equinus position may also lead to only partial weightbearing, which decreases venous circulation.^[10]^ We therefore suggest prospective randomized trials to examine leg immobilization with less rigid plantar flexion and more mobility in the ankle joint compared to equinus positioning in relation not only to blood flow but also to the risk of venous thromboembolism.

Whether poor AD caused an increased risk of DVT or whether DVT triggered, for example, increased swelling leading to poor AD, is unclear from the present data. The observation that increased calf circumference was neither associated with ankle dorsiflexion nor the risk of DVT, however, indicates that the asymptomatic DVTs did not result in swelling of the calf and is therefore in line with the concept that poor AD preceded the DVT. The finding that calf circumference was not associated with the risk of DVT is of importance since increased calf circumference is 1 criterion of Wells score for diagnosing the risk of DVT.^[7]^ Thus, ankle dorsiflexion may be a more prognostic measure of DVT than calf circumference in patients with leg immobilization. This conclusion, however, warrants further research.

The results from the multiple linear regression analyses confirmed the association between ankle dorsiflexion and risk of DVT when taking patient characteristics into account. This analysis furthermore demonstrated that the odds of a DVT were 3 times higher with a poor ankle dorsiflexion than the odds for a DVT with good ankle dorsiflexion. Such a high odds ratio, equal to the odds ratio of DVT in patients over compared to under the age of 40, suggests that it may be worthwhile to further study ankle dorsiflexion after limb immobilization as a prognostic marker for the risk of DVT.^[6]^

The second main finding of this study pertains to the fact that patients with greater ankle dorsiflexion after plaster cast removal exhibited better patient-reported outcome at 3 months postoperatively. The observation of less pain in the limb at 3 months in patients with greater ankle dorsiflexion at 2 weeks, in both uni- and multivariate statistics, suggests that a plaster cast with less equinus position and more flexibility may result in less early pain in the limb. The observation that ankle dorsiflexion did not affect the patient-reported outcome at 1 year suggests that the early increased experience of pain related to decreased ankle dorsiflexion does not produce any long-term subjective disabilities. It has earlier been shown that pain at 3 months after ATR surgery was associated with poor functional outcome at 1 year assessed with the calf muscle endurance test.^[20]^ The patients with poor AD, however, were not affected in their functional outcome at 1 year as assessed with the calf muscle endurance (heel-rise) test.

Our finding of ankle dorsiflexion not associated with the heel-rise height suggests that no major tendon lengthening occurred in the patients with greater ankle dorsiflexion. Less heel-rise height in patients with ATR repair has earlier been shown to correlate with tendon elongation, which is a limiting factor for optimal limb function.^[17]^ Our conclusion that increased ankle dorsiflexion is not associated with tendon lengthening is supported by a meta-analysis comparing bracing and casting, which demonstrated no complications or tendon elongations in the bracing group.^[12]^

A potential limitation of this study is that the underlying factors relating to increased ankle dorsiflexion are not further known. AD could presumably be due to more surgical tension of the tendon or genetic differences leading to a plaster cast in more plantar flexion. Other factors affecting the amount of ankle dorsiflexion directly after the removal of the plaster cast could be pain due to more or less motion within the plaster cast, a DVT, fear of movement/re-rupture, pull in the wound/scar, discomfort, and personality. To counteract some of these aspects the patients performed standardized active exercises before the measurements and were assured that this movement was safe for the tendon.

The strengths of our study are that all patients underwent the same study protocol, regarding local anesthesia and surgical techniques, as well as a meticulous assessment of DVT and AD. The study was also well enough powered to establish differences in ultrasound diagnosed DVTs in relation to AD.

## 5 Conclusions

We therefore conclude that decreased ankle dorsiflexion assessed after removal of lower limb immobilization may be associated with a higher risk of DVT, but not of any long-term impairments of outcome. The results of this observational study warrant further prospective studies to confirm the effects of ankle dorsiflexion on the risk of developing venous thromboses.
